# Targeting Mitochondrial Apoptosis to Overcome Treatment Resistance in Cancer

**DOI:** 10.3390/cancers12030574

**Published:** 2020-03-02

**Authors:** Natalie Yan Li Ngoi, Clarice Choong, Joanne Lee, Gregory Bellot, Andrea LA Wong, Boon Cher Goh, Shazib Pervaiz

**Affiliations:** 1 Department of Haematology-Oncology, National University Cancer Institute, National University Health System, Singapore 119228, Singapore; natalie_yl_ngoi@nuhs.edu.sg (N.Y.L.N.); clarice_choong@nuhs.edu.sg (C.C.); joanne_lee@nuhs.edu.sg (J.L.); Andrea_LA_WONG@nuhs.edu.sg (A.L.W.); boon_cher_goh@nuhs.edu.sg (B.C.G.); 2 Department of Hand & Reconstructive Microsurgery, University Orthopedic, Hand & Reconstructive Microsurgery Cluster, National University Health System, Singapore 119228, Singapore; gregory_bellot@nuhs.edu.sg; 3 Cancer Science Institute, National University of Singapore, Singapore 117599, Singapore; 4 Department of Physiology, Yong Loo Lin School of Medicine, National University of Singapore, Singapore 117593, Singapore; 5 NUS Graduate School for Integrative Sciences and Engineering, National University of Singapore, Singapore 119077, Singapore; 6 National University Cancer Institute, National University Health System, Singapore 119228, Singapore

**Keywords:** apoptosis, targeted therapy, cancer therapeutics, BCL-2, MCL-1, BCL-xL

## Abstract

Deregulated cellular apoptosis is a hallmark of cancer and chemotherapy resistance. The B-cell lymphoma 2 (BCL-2) protein family members are sentinel molecules that regulate the mitochondrial apoptosis machinery and arbitrate cell fate through a delicate balance between pro- and anti-apoptotic factors. The recognition of the anti-apoptotic *BCL2* gene as an oncogenic driver in hematological malignancies has directed attention toward unraveling the biological significance of each of the BCL-2 superfamily members in cancer progression and garnered interest in the targeting of apoptosis in cancer therapy. Accordingly, the approval of venetoclax (ABT-199), a small molecule BCL-2 inhibitor, in patients with chronic lymphocytic leukemia and acute myeloid leukemia has become the proverbial torchbearer for novel candidate drug approaches selectively targeting the BCL-2 superfamily. Despite the inspiring advances in this field, much remains to be learned regarding the optimal therapeutic context for BCL-2 targeting. Functional assays, such as through BH3 profiling, may facilitate prediction of treatment response, development of drug resistance and shed light on rational combinations of BCL-2 inhibitors with other branches of cancer therapy. This review summarizes the pathological roles of the BCL-2 family members in cancer, discusses the current landscape of their targeting in clinical practice, and highlights the potential for future therapeutic inroads in this important area.

## 1. Introduction—Apoptosis from the Chemotherapy Lens

Chemotherapy resistance in cancer has been attributed to multiple mechanisms, which often act in concert [1,2]. This repertoire includes altering drug transport through influx/efflux pumps such as ATP-binding cassette transporters [3] and P-glycoprotein overexpression [1]. Intracellularly, well-described downstream mechanisms include activation of key signaling pathways, drug-target alteration [4] and repair of drug-induced DNA damage [2]. Extrinsic to the cancer cell, cross-talk between tumor cells with the tumor microenvironment adds to chemo-resistance [5]. Tumor heterogeneity and the existence of cancer stem cells, may further limit treatment response. 

Increasingly, dysregulation of drug-induced autophagy and apoptosis has been recognized as a key mechanism of carcinogenesis and chemotherapy resistance, whereby the surviving cancer cell continues to accumulate oncogenic mutations which further propagate tumor progression [6]. Targeting apoptosis therefore holds promise in overcoming resistance to cancer therapy. Recently, venetoclax (ABT-199) has successfully achieved USA Federal Drug Administration (FDA) approval for the treatment of patients with chronic lymphocytic leukemia (CLL) and acute myeloid leukemia (AML), confirming that apoptosis-targeting strategies have finally come of age. In this article, we discuss the roles of the BCL-2 superfamily in carcinogenesis and treatment resistance, and review the successes and failures of strategies targeting the BCL-2 family members in cancer therapy. 

## 2. The BCL-2 Superfamily and Its Role in Apoptosis

### 2.1. The BCL-2 Superfamily Controls the Intrinsic Apoptosis Pathway

Apoptosis is effected via the intrinsic and extrinsic pathways. The extrinsic, or death-receptor mediated pathway, is initiated when cell death receptors such as Fas, TNFR1, TRAIL-R1, TRAIL-R2, DR3 and DR6, interact with their ligands on the cell surface. Activation of Fas, TRAIL-R1 or TRAIL-R2 leads to the formation of a “death-inducing signaling complex” (DISC) and triggers a cascade of caspase activation culminating in apoptosis, while the activation of other receptors induces apoptosis by triggering different signaling pathways such as NF-κB [7,8]. Detailed discussion of the extrinsic pathway and its targeting is beyond the scope of this review. The intrinsic, or mitochondrial pathway, responds to intracellular apoptotic stimuli such as viral infection, oxidative stress, calcium flux and DNA damage caused by drug or radiation exposure [9,10]. When committed to apoptosis, mitochondria outer membrane permeabilization (MOMP) is the decisive event through which cytochrome c and the second mitochondria-derived activator of caspase (SMAC) are released into the cytoplasm, triggering apoptosome assembly and caspase 9 activation [7]. Downstream executioner caspases 3, 6 and 7 cause cellular dismantlement and cytoskeletal protein degradation, which lead to the classic morphological features of crenation, DNA condensation and, ultimately, cell death [11].

**Figure 1:**
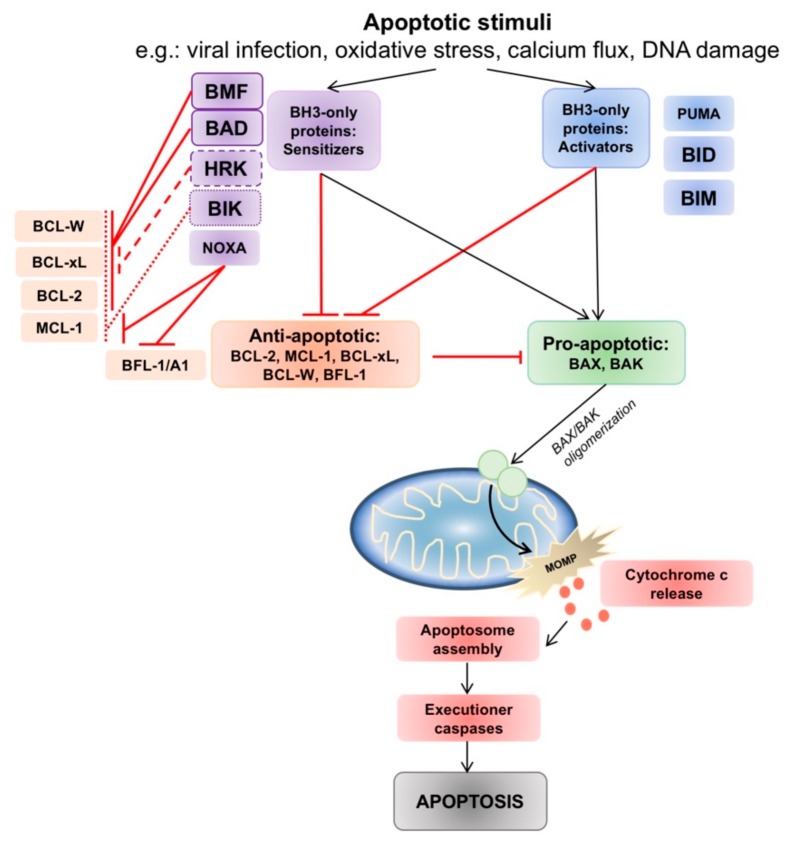
The intrinsic apoptotic pathway and interactions between pro- and anti-apoptotic B-cell lymphoma 2 (BCL-2) family members. Intrinsic pathway apoptotic stimuli such as viral infection, oxidative stress, calcium flux and DNA damage lead to changes in the balance of pro- and anti-apoptotic BCL-2 family members. The anti-apoptotic proteins act to prevent BAX/BAK activation. Activator BH3-only proteins (PUMA, tBID, BIM) inhibit all anti-apoptotic members, whereas sensitizer BH3-only proteins interact and engage selective anti-apoptotic members, allowing BAX/BAK oligomerization and indirect activation. Oligomerization of BAX/BAK in the mitochondrial membrane commits the cell to mitochondria outer membrane permeabilization (MOMP), and triggers a downstream caspase cascade which ends in apoptosis.

The BCL-2 family members are central regulatory players in the intrinsic mitochondrial apoptotic program, and their interplay controls cell fate [12] (Figure 1). More than 25 BCL-2 family members have been identified. Advances in structural resolution of these members have categorized them into three subfamilies—(1) the multidomain anti-apoptotic members (BCL-2, BCL-xL, MCL-1, BCL-w, BCL-B/Boo and BFL-1/A1), (2) the multidomain pro-apoptotic members (BAX, BAK), and (3) the BH3-only members (BAD, BID, NOXA, HRK, BMF, PUMA, BIM). Multi-domain members in (1) and (2) have four BCL-2 homology (BH) domains (BH1, BH2, BH3 and BH4) each, while the BH3-only members are comprised of only a single short BH3 domain [13,14,15]. The BH3 motif is composed of 9 to 15 amino acids and is uniquely conserved across all BCL-2 family members [16]. BH3 interactions are responsible for orchestrating the BCL-2 interactome via a BH3-into-hydrophobic groove mechanism [17,18], which allows the formation of homo- and heterodimers that control apoptotic function [19]. Minor alterations in the amino acid sequences of the binding grooves and BH3 domains control the specificity of these interactions. 

The balance between pro- and anti-apoptotic family members determines if intrinsic apoptosis will proceed. When pro-apoptotic BAX or BAK are liberated, they are able to oligodimerize in the outer mitochondrial membrane, leading to the formation of a mitochondrial membrane pore which commits the cell to MOMP. The BH3-only proteins have complex roles as death sentinels that link apoptotic signals to the intrinsic pathway, and are divided between two roles– either as “direct activators” (tBID, BIM, PUMA) of BAX and BAK, exposing the BH3 domain of BAX and BAK to facilitate oligodimerization [20]; or as “inactivators” or “sensitizers” (BAD, BIK, BMF, HRK, NOXA) by binding with anti-apoptotic BCL-2 thus allowing BAX/BAK to be unrestrained to trigger MOMP [21] (Figure 1).

### 2.2. Regulation of BCL-2 Family Members 

The importance of apoptosis in homeostasis requires that it is tightly regulated. The canonical roles of the BCL-2 subfamilies suggest that apoptosis may be triggered through inactivation of the anti-apoptotic multidomain subfamily proteins, or an increase in concentration of BH3-only proteins. Yet the BCL-2 puzzle has proven far more complex and often unpredictable, contributed by pleiotropic effects of multiple signaling controls, as well as post-transcriptional and post-translational modification processes, in modifying the affinity between BCL-2 family members [16]. For example, PI3K-Akt activation leads to phosphorylation and inactivation of BAD [22], leaving BCL-2 free to inhibit apoptosis, while increasing the expression of anti-apoptotic genes [11]. Similarly, activation of the extracellular signal regulated kinase (ERK) pathway results in increased transcription of the anti-apoptotic subfamily of BCL-2 members, and increased ubiquitination and subsequent degradation of pro-apoptotic members, leading to cell survival [23]. ERK-mediated phosphorylation of MCL-1 at T163 was further shown to stabilize MCL-1, leading to suppression of apoptosis in various hematological malignancy cell lines [24]. 

In the nucleus, genomic alterations such as chromosomal translocations and gene amplifications may increase BCL-2 levels. A notable example would be in CLL, where deletion of chromosome 13q in >50% of patients leads to silencing of the microRNAs miR-15 and miR-16, which are responsible for degrading BCL-2 RNA, resulting in BCL-2 overexpression [25]. Post-translational modifications moderate protein functions through ubiquitination, proteolysis, phosphorylation and proteasomal degradation [26]. Phosphorylation of BCL-2 at S70 [27] has been described to alter its anti-apoptotic ability [28] and confer resistance to taxane chemotherapy [29]. Specific BIM phosphorylation sites have the ability to affect its BCL-2 binding capability, resulting in an anti-apoptotic phenotype, while mutations at other phosphorylation sites (Ser-55, -65 and -73) tag BIM for proteasomal degradation, increasing therapy resistance [30]. Phosphorylation of BAX at specific residues (S184), mediated by Akt activation, has been suggested to switch BAX from pro- to anti-apoptotic in phenotype, by allowing it to sequester activator BH3 proteins [18].

### 2.3. Dysregulation of BCL-2 Family Members in Carcinogenesis and Treatment Resistance

Dysregulation of the anti-apoptotic members BCL-2, BCL-xL and MCL-1 have been widely described in carcinogenesis, cancer progression and chemotherapy resistance [31]. Cancer cells often upregulate anti-apoptotic BCL-2 proteins, thus tilting the ratio of anti- versus pro-apoptotic members to fall in favor of apoptosis evasion, even in the presence of stimuli from chemotherapeutic agents [6]. CLL is considered one of the classical hematological malignancies attributable to failure of apoptosis. Nearly all CLL patients have increased BCL-2 expression. Repression of BCL-2 at the post-transcriptional level allowed for the induction of apoptosis in CLL cell lines [25]. BCL-2 overexpression is a key event in follicular lymphoma (FL), driven by pathological chromosomal t(14; 18) translocation, whereby the *BCL2* oncogene is pathogenically translocated to the immunoglobulin heavy chain (IGHV) gene locus, leading to its amplification. In diffuse large B-cell lymphoma (DLBCL), concomitant overexpression of BCL-2 and MYC is classified as a “double-hit” DLBCL, which is associated with a dismal prognosis, high risk for relapse, resistance to standard chemotherapy and justifies upfront escalation to more intensive treatment. These observations have fueled strategies therapeutically targeting the anti-apoptotic BCL-2 members in cancer treatment.

An interesting and somewhat non-canonical aspect of the functional biology of BCL-2 is ability to maintain a mild mitochondrial pro-oxidant milieu while preventing deleterious levels of reactive oxygen species (ROS) production triggered by oxidative stressors through the regulation of cytochrome c oxidase activity [32]. This mechanism appears to be the result of an interaction between BCL-2 and the subunit COX Va that shifts the ratio of COX Va to COX Vb subunits, thus modulating cytochrome c oxidase activity. The modulation of ROS production by BCL-2 expression is a critical component of its anti-apoptotic activity as cells subjected to oxidative stress inducers modulate their mitochondrial redox metabolism to buffer the excess ROS production, thereby promoting cell survival [33]. In addition, the pro-oxidant milieu generated through superoxide anion production by an increased expression of BCL-2 was shown to be linked to an interaction between BCL-2 and the small GTPase Rac1, a critical regulator of NADPH oxidase, responsible for superoxide production [34]. Interestingly, a mild to moderate increase in intracellular superoxide anion (*pro-oxidant state*) has also been shown to impact the phosphorylation status of BCL-2, specifically at S70 via the generation of peroxynitrite (a reaction product of superoxide and nitric oxide). This involves peroxynitrite mediated nitrative modification of the regulatory subunit B56δ of the protein phosphatase 2A (PP2A), which prevents holoenzyme assembly and results in the sustained S70 phosphorylation of BCL-2 to stabilize its anti-apoptotic activity [35]. These findings provide evidence for an intricate crosstalk between BCL-2 and cellular redox metabolism, thereby delineating a novel facet in the biology of this death regulatory protein with potential therapeutic implications. 

*MCL1* is one of the most highly amplified genes in human cancers [36]. In hematological malignancies, increased levels of MCL-1 have been described in multiple myeloma (MM) [37], DLBCL [38], AML, chronic myeloid leukemia (CML) and mantle cell lymphoma (MCL). Many chemotherapeutic agents affect apoptosis through the reduction of MCL-1 levels. In CLL cell lines, up-regulation of MCL-1 after co-culture with stroma was linked to fludarabine resistance [39]. Conversely, knock-down of *MCL1* in mice models not only triggered apoptosis of transformed AML cells but also salvaged AML-afflicted mice from disease progression [40]. 

Finally, elevated BCL-xL expression has also been observed in MM [41] and non-Hodgkin’s lymphoma (NHL), and is implicated in their progression. In one study, transgenic mice with overexpression of BCL-xL readily developed lymphomas [42]. This is further supported by studies showing that interactions between pro-apoptotic BCL-xL and anti-apoptotic BIM control the apoptosis rate in MYC-related lymphoma [43].

Conversely, the loss of pro-apoptotic proteins appears to be relatively uncommon. Somatic inactivation of *BAX* (and *BAK*) has been reported in both solid and hematological cancers [44]. Deletion or silencing of NOXA, PUMA or BIM expression has been described in the pathogenesis of hematologic cancers and their response to chemotherapy [45,46]. Of note, *BIM* is deleted in 17% of MCL [47], while *BAX* mutations occur in 20% of hematologic cancers such as CLL, FL, MCL and NHL. In mouse fibroblast models, loss of both BAX and BAK led to resistance to chemotherapy-induced apoptosis [48]. Additionally, loss of BAX in colon cancer cells led to 5-fluorouracil resistance [49]. 

Indeed, the complex roles of the BCL-2 family members have created immense potential for targeting. Progressive and stepwise improvements in our mechanistic understanding of apoptosis have since allowed for the identification of entry points into this network, toward the promise of optimal therapeutic targeting in cancer. In the next section, we discuss the historical advancements in BCL-2 family targeting that have led to the success of venetoclax in modern day hematological malignancy treatment, and delve into upcoming novel strategies. 

## 3. Targeting the BCL-2 Superfamily: A Summary of the Current Landscape

### 3.1. Antisense Oligonucleotides (ASO)

ASOs were the first approaches employed for BCL-2 inhibition. These are complementary strands that hybridize with and silence anti-apoptotic BCL-2 subfamily mRNA, leading to hydrolysis of the mRNA and promoting apoptosis [50,51]. Oblimersen is an 18-antisense oligonucleotide complementary to the first six codons of BCL-2 mRNA that was evaluated in a variety of hematological malignancies. Promising response rates were seen when combined with standard chemo-immunotherapy [52,53], and also allowed lower doses of chemotherapy to be administered. Reduced BCL-2 mRNA and protein levels were noted in AML patients who achieved a complete response (CR) with oblimersen, providing proof-of-principle of its mechanism of action [53]. Common toxicities included fever, fatigue, gastrointestinal side effects and night sweats. However, on several phase III studies, no survival advantage could be shown for oblimersen addition [52]. Despite this, several patients treated with oblimersen on study appeared to derive durable benefit from this drug [52]. Other ASOs under evaluation include SPC2996, PNT2258 and bispecific ASOs targeting BCL-2/BCL-xL. 

### 3.2. BH3-Mimetics

The recognition of BH3-only proteins as natural inhibitors of BCL-2 proteins led to the development of BH3-mimetics. These small molecules are homologous to the BH3 domains of anti-apoptotic BH3-only proteins, and bind competitively to the hydrophobic groove of anti-apoptotic proteins, displacing BAX/BAK or pro-apoptotic BH3-only molecules, inducing apoptosis. Venetoclax (ABT-199, Abbvie Inc, North Chicago, IL, USA), which targets BCL-2, was the front-runner inhibitor in developmental pipelines for BH3-mimetics, and its FDA approval across four indications represents a major milestone in this field. To date, BH3-mimetics specifically inhibiting BCL-2, MCL-1 and BCL-xL, respectively are undergoing evaluation. 

#### 3.2.1. Gossypol and AT-101

Gossypol acts as a pan-BCL-2 family inhibitor [54], and has both BAX/BAK-dependent [55] and -independent [56] mechanisms of action. In preclinical studies, gossypol demonstrated promising activity through activation of the intrinsic apoptotic pathway in CML [57], NHL [58] and MM [59]. In-vivo studies in mouse models [60] showed significant slowing of tumor growth when gossypol was combined with CHOP chemotherapy, compared to either CHOP or gossypol alone [60]. However, gossypol has significant off-target side effects including dose-limiting thrombocytopenia, preventing it from advancing into clinical trials. The more potent, and orally-available enantiomer of gossypol, AT-101 progressed further in its development. However, despite promising preclinical data, early phase studies of AT-101 in combination with docetaxel for prostate cancer or non-small cell lung cancer (NSCLC) did not show improved outcomes [61,62] (Table 1). In CLL, AT-101 with rituximab showed only modest efficacy [63]. Gastrointestinal toxicities (such as nausea, vomiting and ileus), fatigue and neutropenia were the most common side effects noted [63]. Newer analogues of AT-101 include TW37 and TM-1206, which have improved affinity to BCL-2, MCL-1 and BCL-xL.

#### 3.2.2. Obatoclax

Obatoclax (GX15-070; Teva Pharmaceutical Industries Ltd, Parsippany, NJ, USA) is a relatively weak polypyrrole pan-BCL-2 family inhibitor that is able to bind to anti-apoptotic BCL-2, BCL-xL, BCL-w, BCL-B, BFL-1/A1 and also MCL-1 with sub-micromolar affinity, allowing BAK/BAX oligomerization and cell death [64]. Obatoclax is also purported to have BCL-2 independent mechanisms, via its effect on the Akt/mTOR signaling pathway [65], which increases the possibility of off-target toxicity. In various hematological malignancy cell lines, and in-vivo mouse models, obatoclax monotherapy showed anti-cancer activity [65,66]. However, raising obatoclax serum levels to clinically effective concentrations in mice models was associated with severe neurotoxicity. Accordingly, when obatoclax was tested in phase I/II trials for AML, CLL, acute lymphoblastic leukemia (ALL), myelodysplastic syndrome, MCL and classical Hodgkin lymphoma (HL) [67,68,69,70], only limited clinical activity was observed (Table 1). Common adverse events included mood disturbances and gastrointestinal side effects, while high grade toxicities appeared to be mainly hematological [68]. Development of obatoclax has been discontinued.

#### 3.2.3. ABT-737

ABT-737 (Abbvie Inc, North Chicago, IL, USA) was the “first-in-class” small molecule inhibitor designed as a BH3 mimetic of BAD. ABT-737 was shown to bind with a much higher affinity (sub-nanomolar concentrations) and more selectively, compared to obatoclax, to BCL-2, BCL-xL and BCL-w [71]. Activity of ABT-737 was shown in MM, and AML cell lines (Table 1). Notably, in CML cell lines, ABT-737 plus imatinib reduced the development of BCL-2 driven imatinib-resistance [72]. The specific binding of ABT-737 to its intended targets resulted in an increase in MCL-1 expression and phosphorylation thus bypassing the effect of ABT-737 and leading to ABT-737-resistance in AML cells [73]. Compounding this, the unfavorable pharmacokinetic profile of ABT-737 further spurred the development of newer generations of BH3 mimetics. 

#### 3.2.4. Navitoclax

Navitoclax (ABT-263; Abbvie Inc, North Chicago, IL, USA) is a second-generation, orally bioavailable BH3-mimetic. Navitoclax binds preferentially to BCL-2, BCL-xL and BCL-w with nanomolar affinity, specifically disrupting BCL-2 and BCL-xL interactions with pro-death BH3 members. However, navitoclax still lacks the ability to antagonize MCL-1 and BFL-1/A1 dependent interactions. In-vivo xenograft models of small cell lung cancer (SCLC), ALL, NHL, MCL and MM showed promising tumor regression [74]. In a phase I study of 29 patients with relapsed or refractory CLL, navitoclax as a single-agent showed an overall response rate (ORR) of 35% in patients receiving a daily dose of at least 110mg, although no CR was observed [75]. Despite this, durable responses >12 months occurred, even in patients with the poor prognostic marker, deletion 17p. Furthermore, a higher ratio of BIM to MCL-1 and BIM to BCL-2 correlated with improved efficacy of navitoclax [75]. As expected, patients with lower pre-treatment MCL-1 levels had improved response to navitoclax. Prominent thrombocytopenia occurred early after treatment-initiation, this was often dose-limiting [75], and consistent with previous data showing that the homeostasis of mature platelets is dependent on BCL-xL. Currently, navitoclax is increasingly under investigation in solid tumors due to the inherent risks of severe thrombocytopenia in patients with hematological malignancies who are already myelosuppressed (Table 1).

#### 3.2.5. Venetoclax: A Selective BCL-2 Inhibitor

Hydrogen bonds between venetoclax and Asp103 on BCL-2 result in the increased selectivity of venetoclax for BCL-2 compared to previous compounds [85]. Venetoclax is an orally-available, extremely potent and selective BCL-2 only inhibitor, and is platelet-sparing [85]. Due to its improved therapeutic window, this drug emerged as the front-runner BH3-mimetic, particularly in malignancies which are BCL-2 dependent. 

As described earlier, the central role of the BCL2 super family in CLL has made this disease a key substrate for studying and developing BCL-2-targeted therapy. In a phase I dose-escalation study of 116 relapsed/refractory CLL and NHL patients treated with venetoclax, ORR of 79% and CR rate of 20% was seen in patients with CLL. This was particularly impressive as the target-population had included heavily-pretreated CLL patients and 90% of patients harbored at least 2 poor prognostic markers, such as chromosome 17p-deletion, 11q deletion, fludarabine-resistance, bulky lymphadenopathy and lack of mutation in IGHV. This potent and rapid cell kill was further confirmed by the unexpectedly high rate of tumor lysis (TLS) in 18% of patients leading to fatalities. Amending the dose schedule to feature a risk-mitigating ramp-up dose, together with monitoring and adequate TLS prophylaxis, helped to prevent this feared side effect. Despite rampant expression of BCL-2 in healthy tissues, other adverse events (AEs) were manageable, such as diarrhea, nausea and neutropenia. Treatment with venetoclax in the dose-escalation cohort resulted in an estimated 2-year overall survival (OS) rate of 84% [86] (Table 2). 

These impressive results led to a pivotal phase II study of 107 patients with relapsed/refractory deletion-17p CLL treated with venetoclax. ORR with venetoclax was 79%, including CR 8%, and responses were seen regardless of the presence of poor-prognostic markers [86] (Table 2). Specifically in CLL patients treated with venetoclax after progressing on the B cell receptor inhibitors (BCRis) ibrutinib or idelalisib, the phase II M14-032 study reported ORR 67% and time-to-response of 2.5 months. Even in a small exploratory subgroup of 28 patients who had previously received more than 1 previous BCRi, encouraging activity was noted [87] (Table 2). 

Venetoclax therapy is made even more convincing by its ability to result in unprecedented phenomenon of undetectable minimal residual disease (uMRD), which is defined when there is <1 CLL cell per 10,000 lymphocytes in marrow or peripheral blood. Low or uMRD has been shown to correlate with improvements in OS [88]. In a pooled analysis of 2 phase II studies of relapsed/refractory CLL patients treated with venetoclax, the PFS rate was 92.8% in patients achieving uMRD at 24 months on treatment [89,90]. The first approval for venetoclax in patients with CLL came in 2016, where patients with 17p deletion were approved to receive venetoclax in the subsequent-line setting. This was later extended to patients with CLL or small lymphocytic lymphoma (SLL) in June 2018, regardless of 17p deletion, in the subsequent-line setting. 

The combination of rituximab, an anti-CD20 antibody, to venetoclax has also shown to be highly effective and able to achieve high uMRD rates in relapsed/refractory CLL. Preclinical data showed that this combination was able to counteract micro-environmental signals that were contributing to venetoclax resistance in CLL [91]. The phase III MURANO study [92] compared venetoclax-rituximab for 6 cycles followed by a 2 year-maintenance treatment, to 6 cycles of bendamustine-rituximab, and showed remarkable improvements in 2-year progression-free survival of 84.9% versus 36.3% [92], as well as 3-year uMRD rate (62% versus 13%) [93]. Another phase III study recently reported results comparing venetoclax-obinutuzumab versus chlorambucil-obinutuzumab in previously untreated CLL patients. Venetoclax-obinutuzumab was associated with significantly improved PFS at 24 months (24-month PFS rate 88.2% versus 64.1%), and this benefit was extended to patients with poor prognostic factors [94]. These impressive results relating to uMRD, together with pooled analysis data suggesting that venetoclax should be sequenced earlier in treatment paradigms, ultimately led to the FDA indication being expanded to all adult patients with CLL or SLL in May 2019. 

Venetoclax monotherapy is modestly active in relapsed/refractory AML. Of note, patients harboring *IDH1/2* mutations appeared to perform better with venetoclax therapy, with CR rate of 33% [95,96]. Further phase Ib studies have also combined venetoclax with hypomethylating agents based on preclinical models demonstrating synergy [97]. When combined with low dose cytarabine, decitabine or azacytidine in untreated elderly patients, CR/CRi rates ranged between 54–68% across studies with a median time to response of 1.2–1.4 months, with tolerable toxicity [98,99] (Table 2). This led to a further FDA breakthrough status in November 2018 for venetoclax in combination with hypomethylating drugs for newly-diagnosed elderly AML patients ineligible for intensive chemotherapy. 

Venetoclax has also shown promising activity in relapsed/refractory MCL. In a phase I trial of 106 patients with relapsed/refractory NHL, patients with MCL had particularly high response rates (ORR of 75%, CR 21% [100]. Venetoclax plus ibrutinib was evaluated on a phase II study, which recruited a majority of relapsed/refractory MCL patients, again showing high response rates of ORR 71%, CR 63% [101], and this is being explored further on a phase III study (Table 2). Venetoclax monotherapy appears to be less active in other NHL, in particular relapsed/refractory DLBCL, where only modest response rates of around 18% were noted [100]. Similarly, in relapsed/refractory MM, ORR for venetoclax monotherapy was 21% [102]. In cell lines, the t(11; 14) (q13;q32) translocation was shown to increase BCL-2:MCL-1 ratio and lead to lower BCL-xL levels, and patients harboring this translocation may benefit the most [103]. 

As alluded to, venetoclax has heralded the way for the development of other BCL-2 inhibitors. Newer BCL-2 inhibitors in the pipeline include S55746 (Servier, Suresnes, France) which has dual BCL-2/ BCL-xL inhibiting capabilities [104,105] (Table 2). It is likely that the role for BCL-2 inhibitors is likely to expand in cancer therapy, and further results are awaited.

#### 3.2.6. BCL-xL—Selective BH3-Mimetics

BCL-xL dependency has been described across tumor types, aggregating mainly in solid tumors [106]. This makes selective BCL-xL inhibition an attractive target, especially in the treatment of venetoclax- resistant cancers. As described earlier, BCL-xL expression in AML, MM and some solid tumor models, is associated with chemotherapy and venetoclax resistance [107,108]. WEHI-539 (The Walter and Eliza Hall Institute of Medical Research, Melbourne, Australia) was the first selective BCL-xL inhibitor published. When bound to BCL-xL, WEHI-539 induced BAK-mediated cell death in SCLC cell lines. When used in osteosarcoma cells that overexpressed BCL-xL, WEHI-539 was able to potentiate the effect of low-dose doxorubicin [109]. However, further development of this compound has been halted due to in-vivo toxicity. On-target toxicities of such inhibitors include thrombocytopenia, which occurs rapidly and reversibly, similar to what was observed with navitoclax. Further BCL-xL selective inhibitors under pre-clinical evaluation include A-1155463 and A-1331852 (Abbvie Inc., North Chicago, IL, USA) (Table 3). 

#### 3.2.7. MCL-1 Antagonists

In healthy tissues, MCL-1 regulates neural and cardiac cell survival. In cancer, not only has the MCL-1 protein been shown to regulate cell survival in myeloid and lymphoid cancers including MM, AML and NHL [36,110], but *MCL1* amplifications have been found in >10% of solid tumor cancer types [106]. In triple-negative breast cancer, *MCL1* amplification correlates with poor prognosis [111]. 

Drug development in MCL-1 inhibition is ongoing with several candidate compounds in early phase testing (Table 3). A-1210477 (Abbvie Inc., North Chicago, IL, USA) was the first inhibitor able to disrupt MCL-1-NOXA and MCL-1-BIM2A interactions selectively [107]. When used in MM and NSCLC cell lines that showed MCL-1 dependency, A-1210477 triggered MOMP and apoptosis [107]. A more potent MCL-1 inhibitor, S63845 (Servier, Suresnes, France) [112], is also undergoing evaluation, and has been shown to have sub-molar affinity to the MCL-1 BH3 binding groove with BCL-2/ BCL-xL binding. In vitro, S63485 induced BAX/BAK-mediated apoptosis in solid tumors, as well as elicited intriguing synergism with tyrosine kinase inhibitors(TKIs) [112]. Several other MCL-1 inhibitors [AZD5991(AstraZeneca), AMG-176, AMG-397(Amgen), S64315/MIK665(Novartis)] are currently undergoing phase I clinical trials in a variety of hematological malignancies (Table 3). Despite these advancements, the concern for the development of side effects of MCL-1-targeting agents on cardiac and neurological systems may pose challenges to clinical development of these agents, and further results are awaited. 

### 3.3. Targeting the BH4 Domain

Similar to BH3, the BH4 domain is conserved amongst the members of the BCL-2 superfamily. Aside from its crucial role in the anti-apoptotic activity of BCL-2, the BH4 domain also is required for other non-canonical functions of the BCL-2 superfamily, such as in calcium homeostasis at the ER [113]. Notably, losing the BH4 domain greatly diminishes the anti-apoptotic function of BCL-2 [114]. Targeting BH4 is therefore emerging as a novel strategy in cancer therapy (Table 3). 

### 3.4. Interference Technology

Interference technologies at the DNA and RNA level utilize a nucleic acid-based approach to block transcription and translation of *BCL2* respectively. Silencing *BCL2* by utilizing RNA interference (RNAi) technology is still in its infancy. Early data regarding the efficacy of this approach have been generated using ALL cell lines and xenografts [115]. PNT2258 (ProNAi Therapeutics Inc., Vancouver, Canada) a first-in-class DNAi drug that consists of a 24-base sequence complementary to regions of DNA that are upstream from sites of gene transcription, thus preventing BCL-2 transcription [116]. In pre-clinical studies, PNT2258 was active in BCL-2 driven xenografts, including in NHL, prostate cancer and melanoma [117]. Differential activity was seen in different NHL cell lines according to their levels of BCL-2 overexpression. Initial phase I studies of PNT2258 confirmed a safe toxicity profile with tolerable lympho- and thrombocytopenia [118]. Initial interesting responses, especially in DLBCL patients, were noted on a phase I study of PNT2258 in relapsed/refractory NHL. However, these results were not corroborated in a phase II study of relapsed/refractory DLBCL, and the development of PNT2258 was subsequently discontinued [119] (Table 3).

## 4. Navigating Anti-Apoptotic BCL-2 Dependency to Tackle Therapy Resistance 

### 4.1. BCL2 Dependency in Intrinsic and Acquired Therapy Resistance

Despite the excellent results obtained from the use of venetoclax, many patients progress after a period of treatment (acquired resistance), while others do not respond at all (intrinsic resistance). Although BCL-2 is overexpressed in a multitude of solid tumors and hematological malignancies, significant responses to venetoclax monotherapy are limited to only a handful of cancer types. This is because, although BCL-2 may be over-expressed, this may not be reflective of its pathological function. Concurrent or dominant expression of MCL-1 or BCL-xL may indicate dependency on these family members instead, leading to intrinsic resistance to and limited utility of venetoclax monotherapy. 

Acquired resistance is also linked to the concept of BCL-2 dependency. Functional redundancy within the BCL-2 superfamily allows acquired resistance to develop by switching reliance on other anti-apoptotic members as a result of treatment pressure. The exact mechanisms of these dynamic inhibitory responses have yet to be defined. In CLL cells, compensatory BCL-xL and BFL-1/A1 upregulation was associated with acquired venetoclax resistance [120,121]. In NHL cell lines, prolonged venetoclax treatment also resulted in increased BCL-xL and MCL-1 expression, mediated by Akt signaling [122]. Similarly, in lymphoma experiments, resistance to ABT-737 was affected through a shift in BCL-2 family member dependency by the upregulation of MCL-1 or BFL-1/A1 [123]. Aberrant NF-kB signaling has been shown to affect resistance to ibrutinib plus venetoclax combination therapy in CLL cells by increasing MCL-1 and BCL-xL expression [124]. 

### 4.2. BH3 Profiling to Define BCL2 Dependency

BH3 profiling is a functional assay technique that has helped to provide clarity on BCL-2 dependency. This technology is able to determine (1) the degree of ‘mitochondrial priming’ of a cell [138], and (2) BCL-2 member dependency, and hence predict response and resistance to therapies targeting the BCL-2 family [139]. 

Briefly, an array of functionally-distinct BH3-only proteins are added to isolated mitochondria or permeabilized cells taken from a fresh cancer sample, and allowed to interact with other BCL-2 superfamily proteins at the mitochondrial surface, inducing MOMP. MOMP is measured indirectly by the amount of cytochrome release or by the loss of inner mitochondrial membrane potential (MMP). In cells that have highly-primed mitochondria, BH3-only proteins rapidly induce MMP loss, compared to cells that have low priming. Importantly, the differing specificity of some BH3-only proteins for anti-apoptotic members e.g.: NOXA for MCL-1 will allow the BCL-2 dependency of the cell to be inferred by the degree of MOMP triggered when different BH3-only proteins are added [140]. 

MM and AML cells with dominant MCL-1 dependency or heterogenous dependency on multiple members—BCL-2, MCL-1, BCL-xL—were predicted to be resistant to BCL-2 inhibitor monotherapy, unless the other members are able to be abrogated [76]. In MM and NSCLC, the presence of MCL-1/BAK complexes predicted sensitivity to MCL-1 inhibition with A-1210477 [107]. The level of mitochondrial priming inferred from BH3 profiling also provides information regarding the depth of response. In CLL cells, high mitochondrial priming pre-treatment was associated with deeper venetoclax responses [141]. In AML cell lines, low mitochondrial priming has been correlated with chemotherapy resistance [142]. 

New developments in BH3 profiling include whole cell JC-1 based technology that allows easier measurements of cytochrome c release through the use of JC-1, a fluorescent probe. FACS technology now allows BH3 profiling to be performed in polyclonal cell populations [120], potentially providing insight into BCL-2 dependency despite tumor heterogeneity. Furthermore, given the described compensatory upregulation of other anti-apoptotic BCL-2 family members such as MCL-1 and BFL-1/A1 in response to ABT-737 treatment in lymphoma [123] and BCL-xL in response to venetoclax in CLL, identification of these changes via BH3 profiling may allow for sequential use of novel inhibitors such as those against MCL-1 or BCL-xL to combat acquired resistance. Therefore, systematic sequential BH3 profiling has promise as a dynamic biomarker to allow us to document changes in a tumor’s anti-apoptotic BCL-2 dependency longitudinally, predict depth of response, and even select the right therapeutic strategy to target specific molecular vulnerability in a personalized approach.

## 5. Expanding Clinical Contexts for BCL-2 Targeting

### 5.1. Promising Combination Strategies in Hematological Malignancies

Despite the gains we have made through venetoclax in specific clinical contexts, rational combinations of BCL2-targeting therapy with chemotherapeutics and other targeted therapy hold promise to advance treatment paradigms. Venetoclax is currently being combined with different branches of cancer therapy in different hematological and solid malignancies, chosen based on known pathways that are aberrant in specific tumor types (Table 2). Aside from combination with chemotherapy, other important combinations under investigation include those with proteasome inhibitors (bortezomib, carfilzomib), PI3K inhibitors (idelalisib), BTK inhibitors (ibrutinib), CDK inhibitors (dinaciclib, palbociclib), MEK inhibitors (cobimetinib), MDM2 inhibitors (idasanutlin) and other novel agents (Table 2). Co-targeting of different BCL-2 family members to overcome resistance, such as concurrent BCL-2 and MCL-1 targeting or BCL-2/ BCL-xL targeting is also under study (Table 2).

The rationale to combine BCL-2 targeted drugs with chemotherapy is based on the understanding of mitochondrial priming. Treatment with BH3-mimetics is expected to raise the mitochondrial priming state, thereby allowing them to act as “chemosensitizers” for synergism with cytotoxic chemotherapy [143] (Table 2). Furthermore, this approach holds benefit not just in enhancing cell kill, but also may reduce treatment doses, thus reducing toxic side effects. 

Venetoclax in combination with BTK inhibitors in CLL and MCL treatment is actively being explored. Samples taken from CLL patients receiving ibrutinib were analyzed in-vitro with the addition of venetoclax, and proved synergism of this combination [144]. Ibrutinib appears to downregulate MCL-1 and BCL-xL, potentiating venetoclax’s effect [144]. Adding venetoclax to obinutuzumab and ibrutinib in combination is being evaluated on a phase Ib study (NCT02427451), and a phase III study (GLOW/CLL3011) is studying ibrutinib plus venetoclax versus obinutuzumab plus chlorambucil (Table 2).

BAK, BAX and other pro-apoptotic members are degraded by ubiquitination and the proteasomal pathway. Therefore, proteasomal inhibition allows for their stabilization and accumulation in mitochondria, increasing the pro- to anti-apoptotic protein ratio [145]. In relapsed/refractory MM treatment, venetoclax, bortezomib and dexamethasone combination therapy initially showed a high ORR. Patients achieving PR or better had higher levels of BCL-2 [129]. However, on the phase III BELLINI study randomizing patients with relapsed/refractory MM to bortezomib combined with venetoclax or matched placebo, although the study showed improved PFS, ORR and uMRD for the venetoclax-containing arm, 13 treatment-emergent deaths occurred in the venetoclax-containing arm. Most deaths were attributable to infection, and this risk strengthened the urge toward a biomarker-driven approach. Authors suggested that this combination could be most relevant in patients with t(11; 14), where a trend towards improved OS was also noted, limiting exposure of toxicity to a smaller group of patients [131,146]. An additional phase II study investigating venetoclax with carfilzomib and dexamethasone (NCT02899052) is underway, interim results describe no new safety signals [130]. 

Strategies inhibiting important cyclin dependent kinases (CDKs) are also promising. CDK9 is a key component of positive transcription elongation factor (pTEFb) which is a transcriptional regulator complex. Inhibition of CDK9 blocks transcription resulting in MCL-1 repression [147]. CDK9 inhibition also down-regulates miRNAs that in turn negatively regulates pro-apoptotic BCL-2 family members, leading to a net activation of pro-apoptotic members. Voruciclib, a CDK1, 4, 6 and 9 inhibitor, synergized with venetoclax in DLBCL models to induce tumor remission [148]. In MM, several pre-clinical studies have similarly described how CDK inhibition down-regulates MCL-1 in cell lines. In AML, inhibition of CDK9 was demonstrated to transcriptionally silence *MCL1*, and thus overcome MCL-1 dependent drug resistance [149]. In venetoclax-resistant AML cell lines and mouse xenografts, voruciclib combined with venetoclax were synergistic in triggering BIM-dependent apoptosis [150]. Several early phase clinical trials investigating combinations of venetoclax with CDK inhibitors are ongoing (Table 2). 

Promising results are also emerging from the combination of PI3K inhibitors (PI3Ki) with venetoclax and other therapies, particularly in CLL. Recent data released from a phase I/II study of umbralisib (a PI3Ki), ublituximab (a CD-20 antibody) and venetoclax in relapsed/refractory CLL included 27 patients, starting with umbralisib-ublituximab debulking to reduce the risk for tumor lysis syndrome, followed by umbralisib-venetoclax starting from the fourth cycle onwards. In 13 patients treated for >7 cycles of triple combination treatment, the ORR was 100% after cycle 7, and in 9 patients who received 12 or more cycles of treatment, 100% of patients attained uMRD. At short follow up of 6.4 months, none of the 27 patients had experienced disease progression [128] (Table 2). 

Finally, novel therapies are being combined with BCL-2 inhibition. MDM2 inhibition has been shown to promote MCL-1 degradation in preclinical AML models [151]. Early results from a phase Ib study combining idasanutlin with venetoclax in relapsed/refractory AML have shown a response rate of 35.9%, with manageable toxicity [152]. Further studies combining venetoclax with novel therapies such as gemtuzumab ozogamicin, enasidenib and liposomal cytarabine and daunorubicin are ongoing (Table 2).

### 5.2. Targeting BCL2 Pathways in Solid Tumor Therapy

Currently, results from targeting the BCL-2 superfamily in solid tumors, using venetoclax or navitoclax have been disappointing [153]. On a wide study of multiple solid tumor cell lines, MCL-1 mRNA was the anti-apoptotic BCL-2 member with the highest levels in glioma, lung, renal, prostate, ovarian and breast cancer lines. In comparison, BCL-2 and BFL-1/A1 mRNA levels were highest in leukemia/lymphoma and melanoma cell lines [154]. This may explain why therapeutic success with venetoclax monotherapy has been thus far limited to hematological malignancies. Strategies targeting MCL-1 in solid tumors, or combinations including MCL-1 could achieve more success [154]. In cervical cancer cell lines, resistance to venetoclax, the BCL-xL selective inhibitor A1331852 or the MCL-1 inhibitor A-1210477 was noted when these agents were used individually. However, combining MCL-1 and BCL-xL inhibitors, or MCL-1 and BCL-2 inhibitors led to inhibition of proliferation in the same cell lines [155]. 

In other solid tumors, BCL-2 pathway targeting could sensitize to standard therapy, possibly related to its effects on mitochondrial priming. In hormone receptor (HR)-positive breast cancer xenografts, the BH3 mimetics venetoclax and ABT-737 potentiated tumor responses to tamoxifen. Further synergy was seen when the BH3 mimetics were combined with PI3K/mTOR inhibitors, which are already approved therapy in HR-positive advanced breast cancer, in addition to tamoxifen [156]. Currently, a randomized phase II study is comparing fulvestrant versus fulvestrant plus venetoclax in advanced HR-positive breast cancer (NCT03584009), and a phase Ib study of combination letrozole, palbociclib and venetoclax in metastatic ER-positive breast cancer is planned (NCT03900884). 

BH3 mimetics were also shown to potentiate chemotherapy efficacy in basal-like HR-negative breast cancer xenografts. Immunocompromised mouse xenografts were treated with either ABT-737, docetaxel or both [157]. As expected, treatment with ABT-737 alone was ineffective, but treatment with combination therapy led to significant improvements in tumor response and OS *in-vivo* in breast cancer xenografts which overexpressed BCL-2. This finding correlated with a marked increase in apoptosis and BIM-BCL-2 dissociation, and suggests a role for BH3 mimetics to sensitize breast cancers to docetaxel chemotherapy. These results further corroborate with *in-vitro* experiments showing that endogenous BCL-2 phosphorylation occurs with spindle poison treatment which then leads to increased endogenous BCL-2/BIM binding. The addition of BCL-2 inhibitors was able to disrupt mitotic BCL-2/BIM binding in-vitro, enhancing paclitaxel cytotoxicity [29]. 

New strategies are exploiting the signaling pathways that induce dependency on BCL-2-like proteins [158]. Oncogenic addiction of a cell to RAS, HER2 or EGFR inhibits apoptosis by downregulating BH3-only activator proteins through the MAPK/ERK pathway [158], however, this may also trigger a second oncogenic signal through MYC which promotes BIM expression. Overall, this may lead to increased BCL-2-like protein dependency and increase sensitivity of oncogene addicted cells to apoptosis induced by BH3 mimetics. Two studies have reported on the upregulation of BCL-2-like members in EGFR-TKI-resistant NSCLC which harbor oncogenic EGFR mutations [159,160]. In one study, erlotinib-resistant EGFR mutant lung cancer cells showed increased MCL-1 expression, and were sensitive to EGFR TKIs when combined with navitoclax [161]. 

In melanoma, low BCL-xL expression was shown to bias the anti-apoptotic pool towards MCL-1. The combination of MCL-1 inhibition using AZD5991 with MEK1/2 inhibitors (MEKi) was noted to induce synthetic lethality by BAX/BAK-dependent cell death in-vivo [162]. AZD5991 was also shown to delay the development of acquired BRAFi/MEKi resistance, and enhanced the efficacy of ERKi in previously-resistant models [162]. Similar observations were made in patient-derived xenograft models of high-grade serous ovarian cancer which were resistant to the MEKi, cobimetinib. Proteomic interrogation showed that cobimetinib upregulated BIM, increasing mitochondrial ‘priming’, and sensitized models to synergistic targeting with the dual BCL-2/X_L_ inhibitor navitoclax [163]. A phase Ib study combining navitoclax with trametinib in RAS-mutant advanced solid tumors is underway (Table 1).

## 6. Future Directions and Challenges

The recognition of the BCL-2 protein superfamily in regulating intrinsic apoptosis has brought attention to its targeting in overcoming treatment-related resistance in cancer therapy. Progressive refinement in the development of selective BCL-2 inhibitors has led to the successful approval of venetoclax, and significant improvement in clinical outcomes of CLL and AML therapy, while minimizing off-target toxicities. This success has catalyzed the progressive development of other BH3 mimetics, which is likely to change practice in the coming decade. Thus far, the limited success seen in other hematological malignancies and solid tumors only serves to underscore the following challenges we face in harnessing the benefit of BCL-2 inhibitors more broadly. 

Firstly, though active development of BCL-xL and MCL-1 inhibitors is ongoing, it is uncertain if these inhibitors will maintain sufficient safety profile for widespread use [164]. Glaringly, no selective BFL-1/A1 inhibitors have been developed, although the ML214 probe may be useful to evaluate potential interaction sites for BFL-1/A1 inhibition [165]. Successful efforts targeting the pro-apoptotic family members are also notably missing from this space; however, apoptotic modulators such as BAM7 which are able to engage the BAX trigger site toward functional oligomerization are under investigation [166]. 

The selectivity of venetoclax has undeniable benefit in allowing off-target toxicity to be minimized. However, this selectivity itself promotes resistance and compensatory upregulation of non-target anti-apoptotic members such as MCL-1, which may necessitate combination or sequential targeting approaches. At the juncture, it remains to be seen if multiple BH3 mimetics can be successfully used in combination due to overlapping toxicity, and clinical trials evaluating the safety of these combinations are underway. 

Thirdly, increasing data is emerging regarding the regulatory role that the mitochondrial membrane itself exerts on the BCL-2 superfamily. Membrane insertion and BAX oligomerization are the rate limiting steps for intrinsic apoptosis to proceed. Changes in the mechanical properties of the mitochondrial membrane may regulate BCL-2 proteins, or the membrane itself may have direct effects in modulating BCL-2 family member function [19]. One study has reported increased resistance for BAX-BCL-xL complexes when membrane inserted, and it is proposed that the inhibition of BAX oligomerization by BCL-2 proteins in the context of cellular membranes may be an effective means to allow the cell to avoid BAX activation [167]. Examining BCL-2 family member interactions in the presence of membranes appears imperative to forward our efforts.

The invention of BH3 profiling technology has made it plausible that a means of examining functional BCL-2 protein dependency and its dynamism during cancer development and progression is now available. Its predictive benefit should be consistently evaluated on prospective clinical trials. Additionally, BH3 profiling was developed in and has immense potential in the current era of BH3 mimetics. However, it is not clear if this technology will also help predict benefit to other anti-BCL-2 therapies such as interference strategies [168]. 

Finally, the BCL-2 family members have numerous non-canonical functions such as cross-talk with metabolic pathways, cellular redox status, involvement in ER calcium homeostasis and autophagy. These pleiotropic effects mean that targeting BCL-2 and BCL-2-like proteins may have a multitude of effects on cancer cell fate, and these consequences on anti-cancer therapy remain under investigation. Recent data have also shown that the BCL-2 family members have an unexpected immunological role, which is provocative for development. In melanoma cells with strong BCL-2 expression, the addition of ABT-737 to co-culture with expanded peripheral blood cytotoxic T-lymphocytes amplified tumor cell kill. As postulated, the addition of BCL-2 inhibitors may sensitize the target tumor cells to perforin/granzyme-B mediated cell kill [169]. More recently, Brokatzky and colleagues have described that activation of BAX/BAK induces mitochondrial DNA release [170], which can go on to trigger the innate immune system through the cGAS-STING signaling pathway. This may have the potential to increase the immunogenicity of immunologically “cold” tumors. In this way, the mitochondrial apoptosis pathway may serve as a modulator of anti-tumor immunity, therefore paving the way for novel combinations of drugs targeting the BCL-2 family together with immune-checkpoint blockade. 

## 7. Conclusions

Despite the exciting advances made in our understanding of the BCL-2 proteins’ role in controlling cell fate and treatment resistance, these observations indicate that the benefit of BCL-2 targeting therapy is not yet fully exploited. The promise of personalized biomarker technology and rational combinations of BCL-2 inhibitors with other branches of cancer therapy are imminent, and will certainly add to our therapeutic arsenal to improve outcomes in a wider group of patients. 

## Figures and Tables

**Table 1:** Early-generation BH3-mimetics inhibiting BCL-2. R/R: relapsed/refractory; CLL: chronic lymphocytic leukemia; HL: hodgkin’s lymphoma; MDS: myelodysplastic syndrome; ORR: objective response rate; FL: follicular lymphoma; FR: fludarabine plus rituximab; iwCLL: international workshop on CLL; MCL: mantle cell lymphoma; CR: complete response; PR: partial response; PFS: progression-free survival; RP2D: recommended phase 2 dose; ALL: acute lymphoblastic leukemia; SCLC: small cell lung cancer; NSCLC: non-small cell lung cancer; OS: overall survival.

Drug	Mechanism of Action	Phase	Treatment Population	Activity	Reference
ABT-737	Binds and neutralizes BCL-2, BCL-xL, and BCL-w.	Preclinical	CLL cell lines	ABT-737 induced CLL cell death in a BAX/BAK dependent manner.	[76,77]
Obatoclax	Inhibits BCL-xL, BCL-2, MCL-1, BCL-w, A1 and BCL-B.	Monotherapy			
		I	R/R HL	No objective responses observed.	[78]
		II	Treatment-naïve MDS	ORR 8%, disease stabilization/response was maintained ≥ 12 weeks in 50%. The study was terminated due to failure to meet pre-specified response endpoint.	[68]
		I	R/R CLL	PR 4%. Dose limiting neurologic toxicities including somnolence, euphoria and ataxia were noticed on study.	[79]
		Combination therapy			
		II	Treatment-naïve FL	Obatoclax single-agent or in combination with Rituximab, no objective responses observed after 12 weeks of single-agent obatoclax.	[80]
		I	R/R CLL	Obatoclax plus FR, ORR was 54% by IWCLL 2008 criteria. Median time to progression was 20 months.	[81]
		I/II	R/R MCL	Obatoclax plus bortezomib was feasible. ORR was 31% (3 CR, 1 PR). Synergy observed in preclinical models was not confirmed.	[70]
Navitoclax (ABT-263)	Targets BCL-2, BCL- X_L_, BCL-w.	Monotherapy			
		I	R/R CLL	PR rate was 35%, median PFS was 25 months. Activity was noted even in patients with chemotherapy-resistant disease, bulky lymph nodes and deletion-17p. Thrombocytopenia was the main dose-limiting toxicity and was dose-dependent. RP2D determined as 250 mg daily.	[75]
		Combination therapy			
		I	R/R ALL	Combination of navitoclax, venetoclax, Peg-asparaginase, vincristine, dexamethasone. ORR 67%.	[82]
		II	Myelofibrosis	Navitoclax and ruxolitinib: ongoing recruitment.	NCT03222609
		Ib	*RAS*-mutant advanced solid tumors	Navitoclax and trametinib: ongoing recruitment.	NCT02079740
Gossypol compounds (AT-101)	Natural phenol derived from the cotton plant. Specific antagonist of BCL- X_L_ and BCL-2.	Monotherapy			
		II	Extensive stage, chemo-sensitive SCLC	Gossypol showed no clinical activity.	[83]
		Combination therapy			
		II	R/R CLL	AT-101 plus rituximab, only PR noted.	[63]
		II	Metastatic NSCLC, second-line therapy	AT-101 plus docetaxel versus docetaxel. No difference in PFS or OS.	[84]
		II	Metastatic castration-resistant prostate cancer	AT-101 plus docetaxel/prednisolone versus placebo plus docetaxel/prednisolone. No difference in OS.	[62]

**Table 2:** Key venetoclax trials including upcoming novel combinations. MRD: minimal residual disease; TLS: tumor-lysis syndrome; CRi: complete remission with incomplete marrow recovery; SLL: small lymphocytic lymphoma; AML: acute myeloid leukemia; IDH2: isocitrate dehydrogenase 2; NHL: non-hodgkin’s lymphoma; DLBCL: diffuse large B-cell lymphoma; WM: waldenstrom macroglobulinemia; MZL: marginal zone lymphoma; MM: multiple myeloma; FCR: fludarabine/cyclophosphamide/rituximab; BR: bendamustine/rituximab.

Phase	Treatment Population	Activity	Reference
Monotherapy			
II	R/R CLL	ORR of 79% was noted, including CR 8%. Grade 4 neutropenia occurred in 23% of patients and were managed with dose reductions and growth factor support.	[90]
II	R/R CLL after progressing on ibrutinib or idelalisib	ORR 67%, rapid time to response.	[87]
I	R/R NHL	Venetoclax monotherapy in MCL, FL, DLBCL, WM and MZL. ORR was 44% and highest in MCL patients (ORR of 75%). Median PFS was 6 months (14 months in MCL patients).	[100]
Ib/II	R/R AML	ORR of 19%, rapid responses were noted with 20% of responders achieving >50% reduction in the percentage of marrow blasts at the first disease assessment.	[96]
Combination therapy – CLL/SLL			
Ib	R/R CLL	Venetoclax and Rituximab: ORR 86% (including CR 51%). 2-year PFS rate was 82%. Negative marrow MRD attained in 57% of patients overall. Clinical TLS occurred in 2/49 patients.	[125]
III	R/R CLL	MURANO: venetoclax-rituximab for 6 cycles followed by a 2 year-maintenance treatment versus 6 cycles of bendamustine-rituximab. Improved 2-year PFS and uMRD rate.	[92]
Ib	R/R CLL	Venetoclax-obinutuzumab for 6 cycles followed by 6 additional venetoclax cycles. 100% of patients achieved uMRD, and 100% PFS at 1 year	[126]
Ib/II	Relapsed and previously untreated CLL	Venetoclax, obinutuzumab and ibrutinib in sequential administration. ORR was 92%, including 42% CR/CRi.	[127]
III	Previously untreated CLL	Venetoclax-obinutuzumab versus chlorambucil-obinutuzumab showed improved 24-month PFS rate favoring venetoclax-obinutuzumab (88.2% versus 64.1%).	[94]
I/II	R/R CLL or Richter’s syndrome	Venetoclax plus duvelisib: ongoing recruitment.	NCT03534323
I/II	Symptomatic CLL	Venetoclax, ublituximab and umbralisib, starting with umbralisib-ublituximab debulking followed by umbralisib-venetoclax from cycle 4 onwards. ORR was 100% after cycle 7. 100% of patients who received ≥ 12 cycles of treatment, attained undetectable MRD.	[128]
III	Previously untreated CLL or SLL	Venetoclax plus ibrutinib versus chlorambucil plus obinutuzumab: active, not recruiting.	GLOW/CLL3011 NCT03462719
III	Previously untreated CLL or SLL without del(17p) or TP53	Venetoclax plus acalabrutinib (AV) versus AV plus obinutuzumab versus chemoimmunotherapy (FCR or BR): ongoing recruitment.	NCT03836261
Combination therapy—AML			
Ib	Untreated older (≥65years) AML, ineligible for intensive chemotherapy.	Venetoclax plus decitabine or azacitidine, CR/CRi: 67%. In patients ≥75 years old or with poor-risk cytogenetics, CR/CRi was 65% and 60% respectively. Median OS was 17.5 months. Treatment was well tolerated.	[98]
I	Treatment-naÏve AML	Venetoclax in combination with intensive chemotherapy: ongoing recruitment.	NCT03709758
I	R/R AML	Venetoclax plus CDK inhibitors alvocidib, CYC065, dinaciclib: all studies ongoing recruitment.	NCT03441555 NCT04017546 NCT03484520
I/II	R/R AML in older (≥60years) patients not suitable for cytotoxic chemotherapy	Venetoclax plus idasanutlin or venetoclax plus cobimetinib: ongoing recruitment.	NCT02670044
I/II	R/R AML with *IDH2* (R140 or R172) mutations	Venetoclax plus enasidenib: planned, not yet recruiting.	NCT04092179
I	R/R CD33+ AML	Venetoclax plus gemtuzumab ozogamicin: planned, not yet recruiting.	NCT04070768
Combination therapy - NHL			
III	Treatment-naïve MCL	Venetoclax plus ibrutinib versus placebo plus ibrutinib: active, not recruiting	NCT03112174
Combination therapy - MM			
Ib	R/R MM	Venetoclax plus bortezomib and dexamethasone. 39% of patients were previously refractory to bortezomib. ORR was 67%, 42% achieved very good PR or better. Patients with high *BCL2* expression had higher ORR compared to patients with low *BCL2* expression.	[129]
II	R/R MM	Venetoclax plus carfilzomib and dexamethasone. Of 17 patients evaluated after completing 2 or more cycles, 3/17 had CR.	[130]
III	R/R MM	BELLINI: Venetoclax plus bortezomib/dexamethasone versus placebo plus bortezomib/dexamethasone. Improved PFS, ORR and MRD for venetoclax arm, however 13 treatment-emergent deaths occurred in the venetoclax-containing arm. Trend towards improved OS in patients with t(11;14). Study suspended for safety.	[131]
I/II	R/R MM	Venetoclax plus daratumumab, bortezomib, dexamethasone: planned, not yet recruiting.	NCT03701321

**Table 3:** Other BCL2 family inhibitors under investigation. SCLC: small cell lung cancer; NSCLC: non-small cell lung cancer; R/R: relapsed/refractory; MM: multiple myeloma; AML: acute myeloid leukemia; NHL: non-hodgkin’s lymphoma; DLBLC: diffuse large B cell lymphoma; ORR: objective response rate; PFS: progression-free survival.

Drug	Mechanism of Action	Phase	Treatment Population	Activity	Reference
**Dual BCL-2/BCL-xL inhibitors**					
S44563	Inhibitor of both BCL-2 and BCL-xL.	Preclinical	Uveal melanoma and SCLC models.	*In-vivo* activity in uveal melanoma and SCLC models.	[104,105]
**BCL-xL -selective inhibitors**					
A-1155463	Selective BCL-xL inhibitor.	Preclinical	SCLC xenografts	More potent against BCL- X_L-_ dependent cell lines compared to WEHI-539. Inhibited SCLC xenograft tumor growth *in-vivo*.	[132]
A-1331852	Selective BCL-xL inhibitor.	Preclinical	Cell lines and xenograft models of seven different solid tumors such as breast cancer, ovarian cancer and NSCLC	Enhances the efficacy of docetaxel *in-vitro* and *in-vivo*.	[107]
**MCL-1 inhibitors**					
AZD5991	Selective MCL-1 inhibitor.	Preclinical	R/R hematological malignancies	Preclinically, preferential activity was noted in hematological cell lines.	[133]
AMG-176	Selective MCL-1 inhibitor.	I	R/R MM and AML	First-in human study, recruitment suspended.	NCT02675452
AMG-397	Selective MCL-1 inhibitor.	I	R/R hematological malignancies	Recruitment suspended due to cardiac toxicity signal.	NCT03465540
S64315/MIK665	Selective MCL-1 inhibitor.	Preclinical and phase I	R/R hematological malignancies	Potent activity *in-vitro* and *in-vivo*. Phase I studies are ongoing.	[134,135] NCT02992483 NCT02979366
**MCL-1 inhibitor plus venetoclax combination therapy**					
AZD5991 plus venetoclax		I	R/R hematological malignancies	Phase I study of AZD5991 in combination with venetoclax: ongoing recruitment.	NCT03218683
S64315 plus venetoclax		I	R/R AML	Phase I study of S64315 in combination with venetoclax: active, not recruiting.	NCT03672695
AMG-176 plus venetoclax		I	R/R AML, NHL, DLBCL	Phase I study of AMG-176 in combination with venetoclax: suspended to evaluate safety.	NCT03797261
**Targeting BH4 domain**					
BDA-366	BCL2 BH4 domain antagonist. Converts BCL-2 into a pro-apoptotic molecule.	Preclinical	MM cell lines and mouse models	BDA-366 inhibited MM tumor growth *in-vitro* and *in-vivo*.	[136]
**BCL2 converting peptides**					
NuBCP-9	20 amino acid peptide that acts as a molecular switch to expose the BH3 domain of BCL-2.	Preclinical	Breast cancer cell lines and Ehrlich tumor mouse models	Synergistic potential of paclitaxel with NuBCP-9 loaded nanoparticles in reducing tumor burden.	[137]
**DNA interference**					
PNT2258	24 base single-stranded DNA oligodeoxynucleotide wrapped in liposomes, inhibits BCL-2 promoter activity.	I	R/R NHL	13 patients were enrolled. Notable responses were observed in DLBCL patients (4/4 DLBCL patients).	NCT01733238
		II	R/R DLBCL	ORR 8.1%; all partial metabolic responses. Median PFS was 1.9 months.	NCT02226965
